# Author Correction: Efficacy of a multidimensional self-management intervention on low-education women with metabolic syndrome: a cluster randomized controlled trial

**DOI:** 10.1038/s41598-023-37968-3

**Published:** 2023-07-03

**Authors:** Shu-Hung Chang, Yi-Ya Chang, Wen-Juei Jeng, Jackson Pui Man Wai

**Affiliations:** 1 Graduate Institute of Gerontology and Health Care Management, Geriatric and Long-Term Care Research Center, Chang Gung University of Science and Technology, Taoyuan, Taiwan; 2 Department of Gastroenterology and Hepatology, Chang Gung Medical Foundation, Linkou Chang Gung Memorial Hospital, Taoyuan, Taiwan; 3 Department of Nursing, Chang Gung University of Science and Technology, Taoyuan, Taiwan; 4 Department of Health Management, Chang Gung Medical Foundation, Taipei Chang Gung Memorial Hospital, Taipei, Taiwan; 5 College of Medicine, Chang Gung University, Taoyuan, Taiwan; 6 Department of Nutrition Science, Fu Jen Catholic University, New Taipei City, Taiwan; 7 Graduate Institute of Sport Science, National Taiwan Sport University, Taoyuan, Taiwan

Correction to: *Scientific Reports* https://doi.org/10.1038/s41598-023-36971-y, published online 26 June 2023

The original version of this Article omitted an affiliation for Yi-Ya Chang. Their correct affiliations are listed below.

Department of Nursing, Chang Gung University of Science and Technology, Taoyuan, Taiwan.

Department of Health Management, Chang Gung Medical Foundation, Taipei Chang Gung Memorial Hospital, Taipei, Taiwan.

The original Article has been corrected.

